# Supplementary figures and images for: Practical indistinguishability in a gene regulatory network inference problem, a case study
Source: ArXiv. 2025 Nov 3:arXiv:2508.21006v2. Originally published 2025 Aug 28. Preprint. [Version 2] (PMC12407701)

## 9 Supplemental Figure

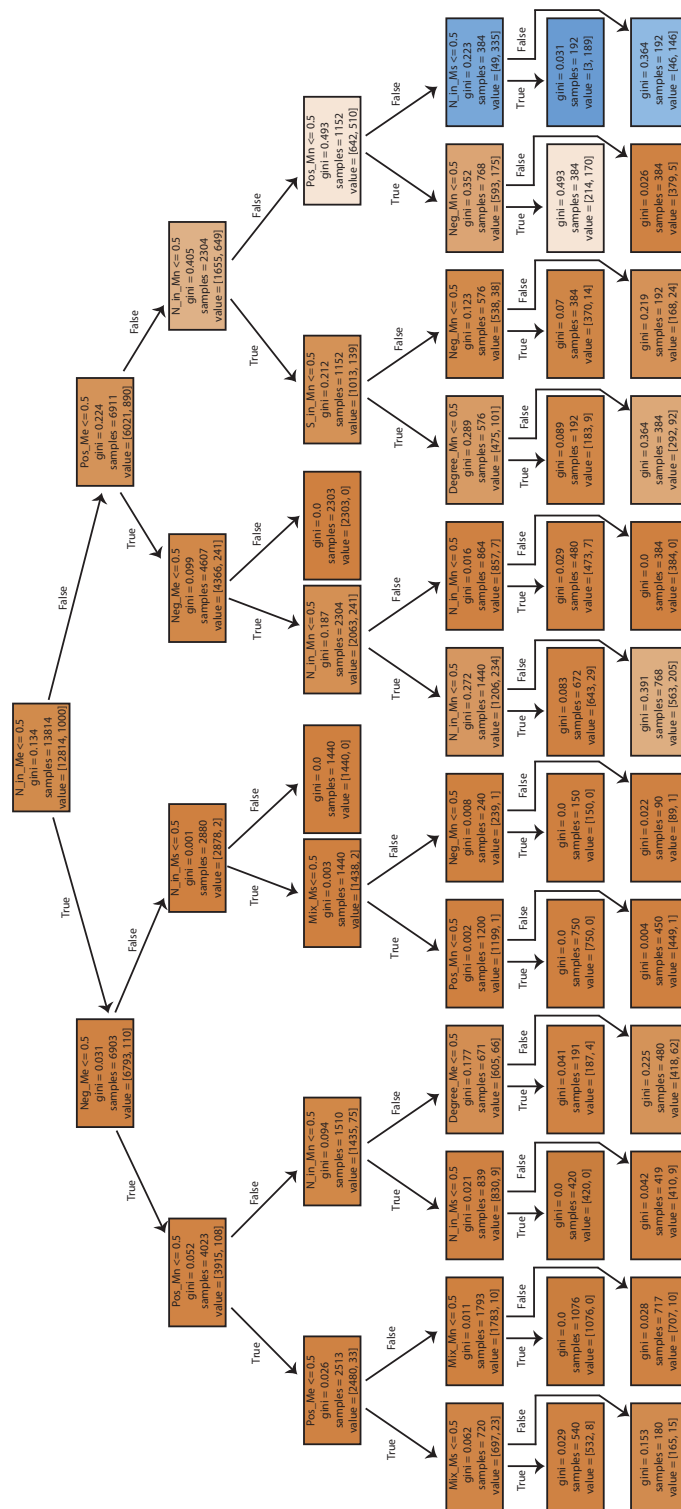

Figure 6: (Caption continued on next page.)

Supplement: Supplement 1 [file NIHPP2508.21006v2-supplement-1.pdf]
